# Supplementary material for: MetaRibo-Seq measures translation in microbiomes
Source: Nat Commun. 2020 Jun 29;11:3268. doi: 10.1038/s41467-020-17081-z (PMC7324362; doi:10.1038/s41467-020-17081-z)
Supplement: Supplementary file 10 — Supplementary Data 7 [file 41467_2020_17081_MOESM10_ESM.zip › File2/Confidence_High_Taxonomy/306564_out.krona.html]

Javascript must be enabled to view this page.

members
magnitude
magnitudeUnassigned
count
unassigned
taxon
rank

306564\_out

60

2
60
superkingdom

phylum
60
976

class
60
200643

171549
60
order

815
60
family

816
genus
60


SRS014613\_contig\_number\_21423SRS017191\_contig\_number\_20538SRS020394\_contig\_number\_9530SRS022137\_contig\_number\_21311SRS043701\_contig\_number\_contig-100\_286.114929SRS048060\_contig\_number\_9500SRS055982\_contig\_number\_19020SRS064232\_contig\_number\_4007SRS064276\_contig\_number\_40412SRS101433\_contig\_number\_15439SRS1041145\_contig\_number\_40932SRS1054691\_contig\_number\_17728SRS1055043\_contig\_number\_21276SRS1055067\_contig\_number\_12528SRS1055099\_contig\_number\_13636SRS893342\_contig\_number\_5445
2292935
species
16


SRS023583\_contig\_number\_6927SRS1041039\_contig\_number\_26852
species
2
821

3
species
817

SRS048981\_contig\_number\_3477SRS078419\_contig\_number\_20215SRS147653\_contig\_number\_7990


SRS013215\_contig\_number\_1160SRS013800\_contig\_number\_2712SRS013940\_contig\_number\_22390SRS013951\_contig\_number\_contig-100\_340.214165SRS015794\_contig\_number\_2733SRS016018\_contig\_number\_16614SRS016381\_contig\_number\_2707SRS017701\_contig\_number\_5285SRS017821\_contig\_number\_15769SRS018049\_contig\_number\_2508SRS019161\_contig\_number\_32239SRS019286\_contig\_number\_2687SRS022524\_contig\_number\_8825SRS023346\_contig\_number\_8481SRS023715\_contig\_number\_contig-100\_213.172480SRS024435\_contig\_number\_30653SRS024549\_contig\_number\_665SRS024663\_contig\_number\_11184SRS042628\_contig\_number\_37893SRS043001\_contig\_number\_8442SRS043768\_contig\_number\_32392SRS045195\_contig\_number\_6348SRS045713\_contig\_number\_12756SRS046717\_contig\_number\_11050SRS052078\_contig\_number\_4684SRS053649\_contig\_number\_10975SRS057717\_contig\_number\_5842SRS075021\_contig\_number\_6108SRS098655\_contig\_number\_10951SRS101268\_contig\_number\_1578SRS1041038\_contig\_number\_contig-100\_39.100183SRS1041136\_contig\_number\_22986SRS1041137\_contig\_number\_15860SRS104636\_contig\_number\_14208SRS140513\_contig\_number\_15762SRS140645\_contig\_number\_contig-100\_57.25854
357276
36
species


SRS014923\_contig\_number\_44879SRS050925\_contig\_number\_17941SRS064757\_contig\_number\_contig-100\_62.141911
3
species
1263042
